# Twisted *vs* Protaper Files in Contemporary Pediatric Endodontics

**DOI:** 10.5005/jp-journals-10005-1244

**Published:** 2014-08-29

**Authors:** Attiguppe Ramasetty Prabhakar, Chandrashekar Yavagal, Rama Krishna Vallu

**Affiliations:** Head, Department of Pedodontics and Preventive Dentistry, Bapuji Dental College and Hospital, Davangere, Karnataka, India; Reader, Department of Pedodontics and Preventive Dentistry, Bapuji Dental College and Hospital, Davangere, Karnataka, India; Postgraduate Student, Department of Pedodontics and Preventive Dentistry, Bapuji Dental College and Hospital, Davangere, Karnataka, India

**Keywords:** Rotary instruments, NiTi, Cutting efficiency, Twisted files, Protaper files

## Abstract

**Objective:** To compare the cutting efficiency of twisted files with protaper files in primary teeth.

**Study design:** It is an experimental, *in vitro* study comparing two groups.

**Results:** The obtained results from the experiment were statistically analyzed with Mann-Whitney U-test. Twisted files showed better cutting efficiency when compared to protaper files.

**Conclusion:** Since twisted files show a better cutting efficiency, they can be efficiently incorporated into the contemporary pediatric endodontic armamentarium.

**How to cite this article:** Prabhakar AR, Yavagal C, Vallu RK. Twisted *vs* Protaper Files in Contemporary Pediatric Endodontics. Int J Clin Pediatr Dent 2014;7(2):93-96.

## INTRODUCTION

Endodontics in children is more challenging and difficult due to the complexities of the root canal system, such as presence of fine and tortuous canals, accessory canals, lateral canals, anastomoses, etc.^1^ Root canal shaping is one of the most important steps in endodontic treatment as it determines the outcome of all subsequent procedures, including chemical disinfection and root canal obturation.^2,3^ Though this stage is adversely influenced by the highly variable deciduous root canal anatomy, it is imperative to achieve complete removal of the vital or necrotic pulp tissue to create sufficient space for irrigation.^3,4^ Furthermore, it is important to preserve the integrity and location of the canal as well as the apical anatomy in preparation for an adequate deciduous root canal filling of the tooth.^3,5,6^ Ever since, the introduction of rotary Nickel-titanium (NiTi) instruments into pediatric endodontics by Barr et al,^8^ NiTi rotary instruments have shown efficiency in achieving optimum root canal shaping with less straightening and more centered preparations of curved primary root canals.^7^ However, a completely different manufacturing process has evolved to introduce the third generation of NiTi rotary instruments into the endodontic market: the twisted file (TF) with R-phase technology with three innovative methods of manufacturing *viz* R-phase heat treatment, metal twisting and special surface conditioning (deoxidation). These processes have shown to increase the instrument resistance, provide greater fexibility and maintain the sharpness of the flutes.^9^ However, no studies have been done on deciduous teeth to support these claims. Hence, this study was performed to compare the cutting efficiency of twisted files with the protaper files in primary teeth.

## MATERIALS AND METHODS

### Study Setting

The present study was conducted in the Department of Pedodontics and Preventive Dentistry, Bapuji Dental College and Hospital, Davangere, Karnataka.

### Study Design

This is an experimental *in vitro* study, comparing between two groups.

### Methodology

The study was conducted on thirty extracted primary teeth with at least two-third roots remaining examples of such teeth are as follows:

- Infected primary molars with considerable bone loss.
- Over-retained primary molars with altered root resorption pattern.
- Primary molars with one root resorbed considerably more than the other, due to altered path of eruption of its successor.
- Infected primary molars with chronic recurrent infection.

**Fig. 1:**
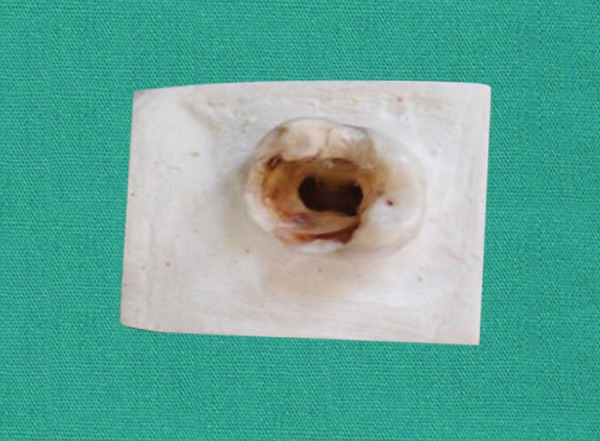
Tooth mounted in dental plaster till the cementoenamel junction

The access opening was done using large round bur (Diaburs, Prime Dental Products, Mumbai, India). The pulp chamber and root canals were irrigated profusely with 1.0% sodium hypochlorite (NaOCl) solution to remove the debris. The no. 10-sized K-file was introduced into each root canal to determine the patency of the root canal. Working length determination: As the #10 file was introduced into the root canal, the tip of the file was seen at the root apex. The final working length was established 1 mm short of this recorded length. RC Prep lubricant was used throughout the procedure. Once the glide path was established, Indian ink was injected into the canals using a 27 gauge needle in all the specimens, and the specimens were left to dry for 30 minutes for proper and complete setting of the ink. Then the teeth were mounted in dental plaster till the cementoenamel junction as shown in Figure 1, so as to standardize the instrumentation procedure. Further, the specimens were divided into two groups containing 15 each.

- *Group I (protaper files)*: Fifteen extracted primary teeth were assigned for protaper files (Dentsply/Maillefer, Switzerland). The manufacturers recommended a speed of 250 rpm in a brushing motion. Canals were irrigated with NaOCl (1.0%) in 5 ml quantity after each instrument, delivered by means of a gauge 27 needle, allowing for adequate back fow. RC Prep (Premier Dental Products) was used throughout the procedure.
- *Group II (twisted files)*: Fifteen extracted primary teeth were assigned for twisted files (SybronEndo). The manufacturers recommended a speed of 500 rpm in pecking motion. Canals were irrigated with NaOCl (1.0%) in 5 ml quantity after each instrument, delivered by means of a gauge 27 needle, allowing for adequate back fow. RC Prep (Premier Dental Products) was used throughout the procedure.

**Fig. 2:**
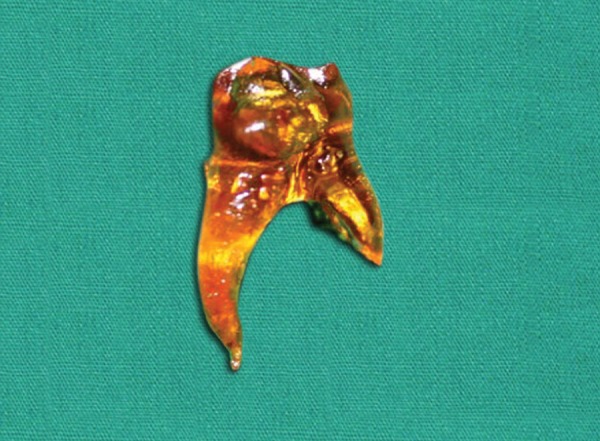
Tooth made clear transparent to analyze the amount of residual Indian ink in the canals

After completion of shaping of the canals with the file, the samples were subjected to decalcification in 10% nitric acid solution and dehydration in different concentrations of ethyl alcohol and immersed in methyl salicylate to make them clear transparent as shown in Figure 2 and then analyzed using stereomicroscope for residual Indian ink in the canals and scored accordingly (Table 1).

Scores given to each root canal are as follows:

- *Score 0*: Total cleaning (no ink remaining in any part of the root canal).
- *Score 1*: Almost complete ink removal (traces of ink found in some areas).
- *Score 2*: Partial ink removal (ink found on some walls in some areas).
- *Score 3*: No ink removal (appreciable amount of ink present).

## RESULTS

**Graph 1:**
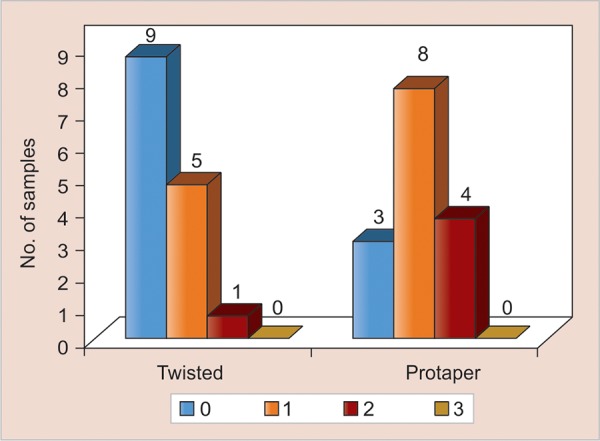
Residual Indian ink scores of both the groups

The residual Indian ink scores of both the groups are shown in Graph 1.

Nine out of 15 samples from twisted file group had shown score 0 and three out of 15 samples from protaper group had shown score 0.

There was significant difference between the two groups with respect to cutting efficiency.

## STATISTICAL ANALYSIS

Statistical analysis was done by SPSS 17.0 and Mann-Whitney U-test.

## DISCUSSION

The main objective of root canal instrumentation is to shape and clean the root canal system effectively while maintaining the original configuration of the canal.^10^ It also aims to create a tapered funnel-shaped preparation with uniform increase in the diameter from the endpoint to the canal orifice so as to facilitate effective irrigation and a three-dimensional obturation of the root canal space.^11^

Since the introduction of rotary NiTi instruments to pediatric dentistry by Barr et al^8^ in 2000, a variety of NiTi instruments have been tried for performing endodontic therapy on primary teeth.

However, the cutting ability of such rotary instruments is a complex relationship of different parameters, such as the cross-sectional design, chip-removal capacity, helical and rake angle, metallurgical properties and surface treatment of the instruments.^12^

**Table 1:** **Table 1:** Mean and median scores of residual Indian ink in the root canals of each group

*Groups*		*No.*		*Score*								*Mean* ± *SD*		*Median*	
				*0*		*1*		*2*		*3*					
				*n (%)*		*n (%)*		*n (%)*		*n (%)*					
Twisted		15		8 (53.3%)		6 (40%)		1 (6.7%)		–		0.5 ± 0.6		0	
Protaper		15		2 (13.3%)		9 (60%)		4 (26%)		–		1.1 ± 0.6		1	

p = 0.02 (p < 0.05), significant

In the present study, a comparison between NiTi instruments produced by a new manufacturing process, the twisted method (TF) with R-phase technology *vs* a well-known and studied instrument produced with the traditional NiTi grinding process, protaper, was performed.

As far as the efficiency is concerned, the present study showed that an irregular amount of dentin was removed by the protaper files which could be explained by their aggressive cutting action.^15^

This implies that the protaper files remove more tooth structure than TF in curved canals.^13^ This could probably be related to the convex triangular cross-sectional design of protaper instruments coupled with a flute design that combines multiple tapers within the shaft of up to 19%. In contrast, TF instruments used in the present study had a constant taper of a maximum 8%^9^ which possibly rendered more even and uniform removal of dentin from the canals.

Previous studies which have also reported more even and uniform removal of dentin, attribute it to the instrument’s high fexibility^13^ and surface deoxidation.^14^

In the present study, root canal instrumentation of primary teeth showed significant differences in the cutting ability between the two systems which were investigated, signifying a more even and uniform removal of dentin with the TF system.

However, more extensive studies are needed to arrive at a superior evidence to support the regular use of twisted files over protaper files for rotary endodontics in primary teeth.

## CONCLUSION

From the present study, it can be concluded that twisted files exhibit a superior cutting efficiency in root canals of primary teeth in comparison to protaper file system.
